# Data driven high resolution modeling and spatial analyses of the COVID-19 pandemic in Germany

**DOI:** 10.1371/journal.pone.0254660

**Published:** 2021-08-18

**Authors:** Lennart Schüler, Justin M. Calabrese, Sabine Attinger

**Affiliations:** 1 Institute of Earth and Environmental Sciences, University Potsdam, Potsdam, Germany; 2 Dept. of Computational Hydrosystems, UFZ—Helmholtz Centre for Environmental Research, Leipzig, Germany; 3 Center for Advanced Systems Understanding (CASUS), Görlitz, Germany; 4 Helmholtz-Zentrum Dresden Rossendorf (HZDR), Dresden, Germany; 5 Dept. of Ecological Modelling, UFZ—Helmholtz Centre for Environmental Research, Leipzig, Germany; Frankfurt Institute for Advanced Studies, GERMANY

## Abstract

The SARS-CoV-2 virus has spread around the world with over 100 million infections to date, and currently many countries are fighting the second wave of infections. With neither sufficient vaccination capacity nor effective medication, non-pharmaceutical interventions (NPIs) remain the measure of choice. However, NPIs place a great burden on society, the mental health of individuals, and economics. Therefore the cost/benefit ratio must be carefully balanced and a target-oriented small-scale implementation of these NPIs could help achieve this balance. To this end, we introduce a modified SEIRD-class compartment model and parametrize it locally for all 412 districts of Germany. The NPIs are modeled at district level by time varying contact rates. This high spatial resolution makes it possible to apply geostatistical methods to analyse the spatial patterns of the pandemic in Germany and to compare the results of different spatial resolutions. We find that the modified SEIRD model can successfully be fitted to the COVID-19 cases in German districts, states, and also nationwide. We propose the correlation length as a further measure, besides the weekly incidence rates, to describe the current situation of the epidemic.

## Introduction

The SARS-CoV-2 virus was first detected in China in late 2019, and then rapidly spread around the world. By March 2020, COVID-19, the disease caused by SARS-CoV-2, was officially declared a pandemic by the World Health Organization [1]. To date, the pandemic has resulted in devastating consequences to life, health, and national economies. The novelty of the SARS-CoV-2 virus, coupled with the comparative lack of clinical research on coronaviruses in general, has left Non-Pharmaceutical Interventions (NPIs), such as masks, lockdowns, and social distancing measures, as the main weapons in the fight against COVID-19. Indeed, NPIs have so far played an important role in modulating the dynamics of the pandemic [2].

In Europe and other regions, NPIs during the first wave of COVID-19 were typically implemented at the national level or at the state level in some federations. In Germany for example, the first COVID-19 case was reported on 2020–01-27 and the first NPIs were imposed on 2020–17-03, with a lockdown of most public places, including school closures. This was followed two weeks later by a ban on meeting with too many people outside of one’s own household, and the number of people simultaneously allowed in supermarkets was restricted. These measures were largely effective [3], and the first COVID-19 wave peaked in Germany at the beginning of April 2020. Relaxations of the nationwide NPIs began by the third week of April, and by May 2020, the first wave in Germany was effectively over. While this type of broad-scale NPI deployment strategy was successful, it was also extremely costly and brought with it many unintended consequences. For example, schools and universities across Germany were completely closed during the lockdown [4]. Additionally, the price and calendar adjusted GDP shrank by 9.7% in the second quarter of 2020 relative to the same period in 2019 [5].

Europe is currently engulfed in a second wave of COVID-19, and despite many advances since the first wave crested, definitive solutions, such as sufficient vaccination capacity, remain elusive. At the same time, the devastating economic, social, and political consequences of nationwide lockdowns have become increasingly apparent. Uncoordinated smaller scale measures failed to keep the virus in check in the fall of 2020. The result has been the reimplementation of nationwide lockdowns. On the one hand, this failure could be interpreted as evidence against the efficacy of local measures. On the other hand, it provides an opportunity to develop more comprehensive strategies for applying NPIs at different scales (e.g., local, regional, national), and for identifying the conditions which require ramping control efforts up to larger scales.

It is therefore imperative that we learn as much as possible about the scale-specific effects of strong NPIs from the first COVID-19 wave. A key limitation is that many analyses so far have focused on the national German level [3, 6], and thus have not been able to resolve local trends. An example for such a local or regional trend is the city of Jena which was the first district to implement mandatory mask-wearing. This measure seems to have effectively and very early stopped the disease [7]. Another example is the largest superspreader event in Germany to date in a meat processing plant, which mainly affected only two districts [8]. Here, we leverage data from the Robert Koch Institute (RKI) [9], reported for each of the 412 administrative districts (i.e., counties) in Germany, to quantify local effects of NPIs from the first COVID-19 wave and the time immediately thereafter. Specifically, we fit modified SEIRD-class compartment models to the RKI data at the district level, and quantify changes in the estimated contact rate for each district across time periods defined by the start and end dates of the various NPIs that were implemented. So far, the studies that have modeled the COVID-19 epidemic in Germany on a district level have focused on evaluating the predictive capabilities of the model itself [10–12]. Whereas we use the more granular data to also facilitate the analysis of the dynamics of spatial patterns of infection clusters, which can yield additional insights into how COVID-19 in Germany responded to NPIs. Finally, our framework also permits a direct, multiscale comparison to highlight how the inferences about NPI effectiveness that can be gleaned depend on the scale of analysis.

## Materials and methods

In Germany, the RKI is responsible for gathering and publishing data on COVID-19. Germany is divided into 401 districts, of which one is Lake Constance and has no residents. The RKI further divides the most populous district of Berlin into its 12 boroughs. For simplicity, these 412 areas for which the RKI publishes data will be called districts from now on. The German reporting obligation of all positive COVID-19 tests to the RKI and the fact that these data are published on the district level makes it possible to model the epidemic at this comparatively high spatial resolution. The population size of the districts is taken from the Federal Statistical Office of Germany [13].

The COVID-19 epidemic in Germany is modeled using a compartmental epidemiological model [14] on the district level. Within each district, the population is divided into ***S**usceptible*, ***E**xposed*, ***I**nfectious*, ***R**ecovered*, and ***D**ead* compartments, with the total population being the sum of the individuals in the compartments minus the COVID-19 related deaths *N* = *S* + *E* + *I* + *R*. To keep the number of parameters as low as possible, the exposed individuals and the asymptomatic cases are handled together in one compartment. The modified SEIRD model is formulated as

$$\begin{array}{cc} \dot{S} & =-\frac{\beta_{j}}{N}IS \end{array}$$ (1)

$$\begin{array}{cc} \dot{E} & =\frac{\beta_{j}}{N}IS-\left(\alpha +\kappa \right)E \end{array}$$ (2)

$$\begin{array}{cc} \dot{I} & =\alpha E-(\gamma +\mu )I \end{array}$$ (3)

$$\begin{array}{cc} \dot{R} & =\kappa E+\gamma I \end{array}$$ (4)

$$\begin{array}{cc} \dot{D} & =\mu I \end{array}$$ (5)

**Fig 1:**
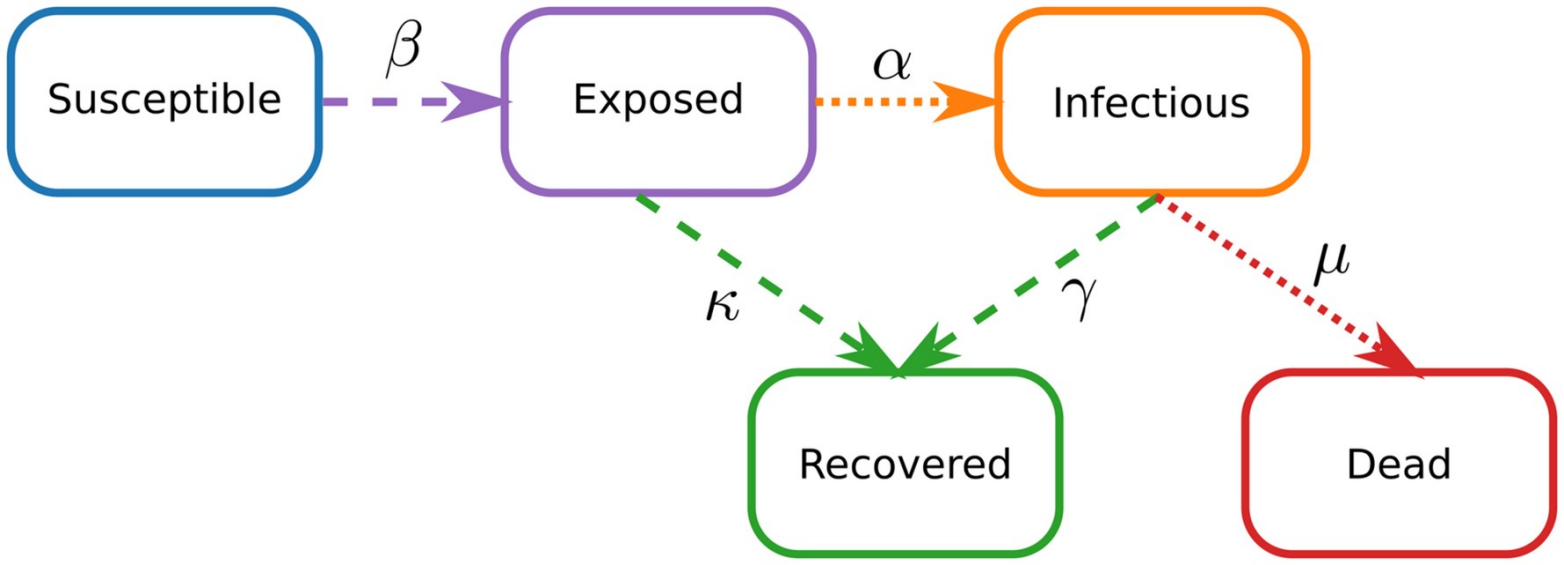
The model structure. A visual representation of Eqs (1)–(5), with the different compartments shown as boxes and the transfer rates as arrows. The data gathered by the RKI are shown as dotted arrows, instead of dashed ones. The color coding of the different compartments is kept consistently throughout this manuscript.

It is assumed that the asymptomatic cases can recover, but not die due to COVID-19, thus Eq (5) is only coupled to Eq (3). A graphical visualization of the system of Eqs (1)–(5) is shown in Fig 1.

The NPIs are modeled by a piecewise constant contact rate *β*(*t*), which is allowed to change at the dates of the NPI implementations. Without loss of generality, this assumption is reformulated to constant contact rates *β*_*j*_, with *j* = 1, 2, …, *M* + 1 and *M* being the total number of NPIs. *β*_*j*_ is exchanged by *β*_*j*+1_ at the date of the *j*-th NPI. All simulations start on the 2020–03-01 and for the initial conditions, we use the number of laboratory-confirmed cases per day *I*_obs_, gathered by the RKI. It translates to the SEIRD-model (1)–(5) as $I_{\text{obs}}\;\hat{=}\;\alpha E$. Thus, the initial condition for the *Exposed* compartment is *E*(0) = *I*_obs_/*α*. For the *Infectious* compartment, the reported cases over the last 1/*α* days are integrated $I\left(0\right)=\int_{-1/\alpha }^{0}I_{\text{obs}}\left(t\right)dt$. This is only an approximation, but it quickly becomes irrelevant, as the compartment is filled by the *Exposed*. The *Recovered* are set to *R*(0) = 0, exactly like the *Dead* *D*(0) = 0, as there where no reported deaths at the initial time. Then, the initial condition for the *Susceptible* compartment is calculated as *S*(0) = *N* − *E*(0) − *I*(0).

Because the latent and asymptomatic cases are lumped together into one compartment, parts of the model structure and some of its parameters cannot easily be mapped to quantities which can actually be measured, like the mean time it takes for the asymptomatic cases to recover. This decision was made in order to keep the number of parameters as low as possible, but at the same time, to have a model, that is flexible enough to reproduce the course of the COVID-19 epidemic across different scales and all districts in Germany.

The assumptions made for SIR-type models break down for small populations. Because the number of cases per day is often already low on the district level without separating the cases into different age groups, we neglect the age distribution of the population to avoid further reducing the number of individuals in the respective compartments.

Using the next generation matrix approach [15], the reproduction number for the SEIRD-model can be calculated yielding

$$\begin{array}{c} R_{j}=\frac{\alpha \beta_{j}}{\left(\alpha +\kappa )(\gamma +\mu \right)} \end{array}$$ (6)

The system of non-linear ordinary Eqs (1)–(5) is numerically solved using an explicit Runge-Kutta method of order 5(4), derived by Dormand et al. [16] and implemented by Virtanen et al. [17].

The *M* + 5 unknown parameters *θ* = (*α*, *β*_1_, *β*_2_, …*β*_*j*_, *γ*, *κ*, *μ*)^*T*^ per district in Eqs (1)–(5) are estimated using Bayesian inference. For the evidence, the number of laboratory-confirmed cases per day *I*_obs_ and the number of deaths related to COVID-19 per day *D*_obs_, gathered by the RKI, are used. These data are grouped together as *X*_obs_ = (*I*_obs_, *D*_obs_)^*T*^. Translating *X*_obs_ to the SEIRD-model (1)–(5), the rate of positively tested cases per day is expressed as $I_{\text{obs}}\;\hat{=}\;\alpha E$ and the rate of COVID-19 related deaths as $D_{\text{obs}}\;\hat{=}\;\mu I$, with *X* = (*αE*, *μI*)^*T*^. Assuming Poisson error distributions, we maximize its log-likelihood

$$\begin{array}{c} L=\sum_{i=1}^{t}(X_{i}\ln\left(X_{{\text{obs}}_{i}}\right)-\ln(\Gamma (X_{i}+1))-X_{i}) \end{array}$$ (7)

with *t* being the total number of days simulated.

The parameter inference is set up for all of the 412 districts and the sampling is repeated 200000 times for each of them. The prior distributions of the parameters are uniform *P*(*θ*)∼*U* and the sampling is done using the Metropolis-Hastings MCMC algorithm [18, 19]. The first 10% of the simulations are used for classical Monte Carlo sampling for the burn-in period. From this, the best parameter set is used as the initial parameter set for the Metropolis sampler. 10 MCMC chains are used for convergence checks. SPOTPY [20] is used for the implementation of the parameter inference.

The RKI gathers and updates its data on the COVID-19 epidemic once a day. These data are downloaded and preprocessed in order to use it for the evidence in the Bayesian framework. Next, the parameter inference is executed for all districts in parallel. Finally, the analyses are done and the plots are created. All these steps are part of a fully automatised workflow on the HPC Cluster EVE [21] at the UFZ Leipzig.

For a comparison with the much more common approach of modeling an epidemic on a national level, the results from all fitted district level simulations are aggregated, first to the level of states within Germany, and subsequently to the national level. This yields three different spatial resolutions that can then be compared: 1) district, 2) state, and 3) national. Additionally, the same SEIRD-model (1)–(5), which was applied to the districts, is also parametrized for the national case and death rates for resolution 3) and for the 16 individual German states for resolution 2).

We performed sensitivity analyses to better understand the model behavior using the extended Fourier amplitude sensitivity test (FAST) algorithm [22]. This method is a variance-based global sensitivity analysis taking parameter interactions into account and is implemented by SPOTPY [20].

The relatively high spatial resolution of German districts makes it possible to use geostatistical methods to identify spatial correlation structures. The variogram is a function describing the type, length, and strength of these spatial correlations. In the cases considered here, the variogram increases monotonically from 0 until it flattens out when it reaches its maximum, which is equal to the variance of the field. The greater the variogram at a certain distance, the smaller the correlations at that distance. If only few and spatially separated superspreader events take place in Germany, we expect to see a high correlation length but a low correlation strength, because all the districts with low infection numbers are highly correlated over a large area (left panel in S1 Fig). But if a superspreader event causes a spreading of infections to neighboring districts and a map of the case numbers on a district level would be plotted, this map would look very patchy, with clusters of high case numbers next to clusters of low case numbers (right panel in S1 Fig). This would be reflected in a variogram with shorter correlation lengths and a higher correlation strength. The variogram is calculated by first computing the pairwise distances of all data points *z*(**x**_*i*_) (in this case the number of positively tested individuals at the centroid **x**_*i*_ of the district in which they where reported). Depending on these pairwise distances ‖**x**_*i*_ − **x**_*j*_‖, the values are grouped into bins of different distances *r* with *r*_*k*_ ≤ ‖**x**_*i*_ − **x**_*j*_‖<*r*_*k*+1_ being the *k*th bin. Now, we define *N*(*r*_*k*_) as the set of all pairwise data points which are grouped into the *k*th bin. By summing the squared differences of all pairs for each bin, the variogram can now be calculated by following equation [23, 24].

$$\begin{array}{c} \gamma \left(r_{k}\right)=\frac{1}{2|N(r_{k})|}\sum_{i,j\in N(r_{k})}{\left(z\left(x_{i}\right)-z\left(x_{j}\right)\right)}^{2} \end{array}$$ (8)

The variograms are calculated and estimated with GSTools [25]. For the calculation of the variograms, the reported cases in each district are accumulated over the time periods of all NPIs separately. This yields a total number of reported cases per district for each NPI period. Then, an empirical variogram is calculated and a variogram model is fitted to it for each of these periods. For all empirical variograms, the best fit was achieved with an exponential variogram model

$$\begin{array}{c} \gamma_{\;e}\left(r\right)=\sigma^{2}\left(1-\exp\left(-r/\lambda \right)\right) \end{array}$$ (9)

with *σ*^2^ being the correlation strength or simply the variance and λ being the correlation length (see lower panel of S1 Fig for an example). A different and commonly used length scale is the percentile scale, which is defined as the distance in which a certain percentage of the variogram’s maximum value (the variance of the field) is reached.

## Results

**Fig 2:**
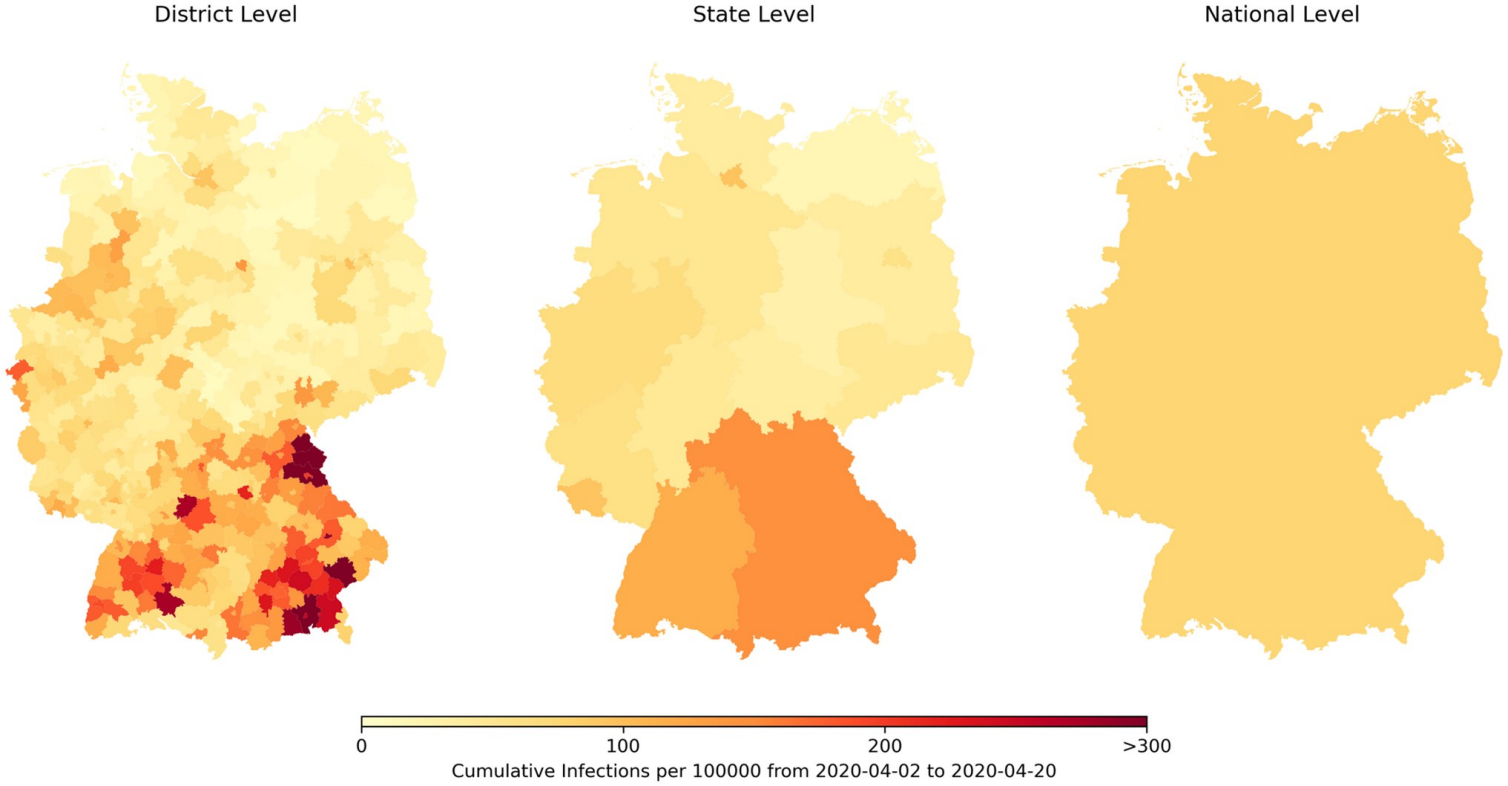
Cummulative reported COVID-19 cases on different hierarchical levels. The number of laboratory confirmed COVID-19 cases per 100000 accumulated from the second NPI on 2020–04-02 until the third NPI on 2020–04-20 on three different spatial resolutions according to the hierarchical administrative divisions of Germany. Borders republished from [32] under a CC BY licence, with permission from GeoBasis-DE / BKG, original copyright 2019. © GeoBasis-DE / BKG (2021).

Visualizing the cumulative reported cases exemplarily for the period of the second NPI on 2020–04-02 to the third NPI on 2020–04-20 on a national, a state, and a district level in Fig 2 shows that reported cases are distributed very inhomogeneously. On the state level one can see that there is a gradient from south to north, but that most of the cases are only reported in relatively small areas can only be seen on the district level. These three scales open up the opportunity of comparing the epidemic over very differently sized populations. The districts have a typical population size in the order of 10^5^, the states of 10^7^, and the nation of 10^8^.

**Fig 3:**
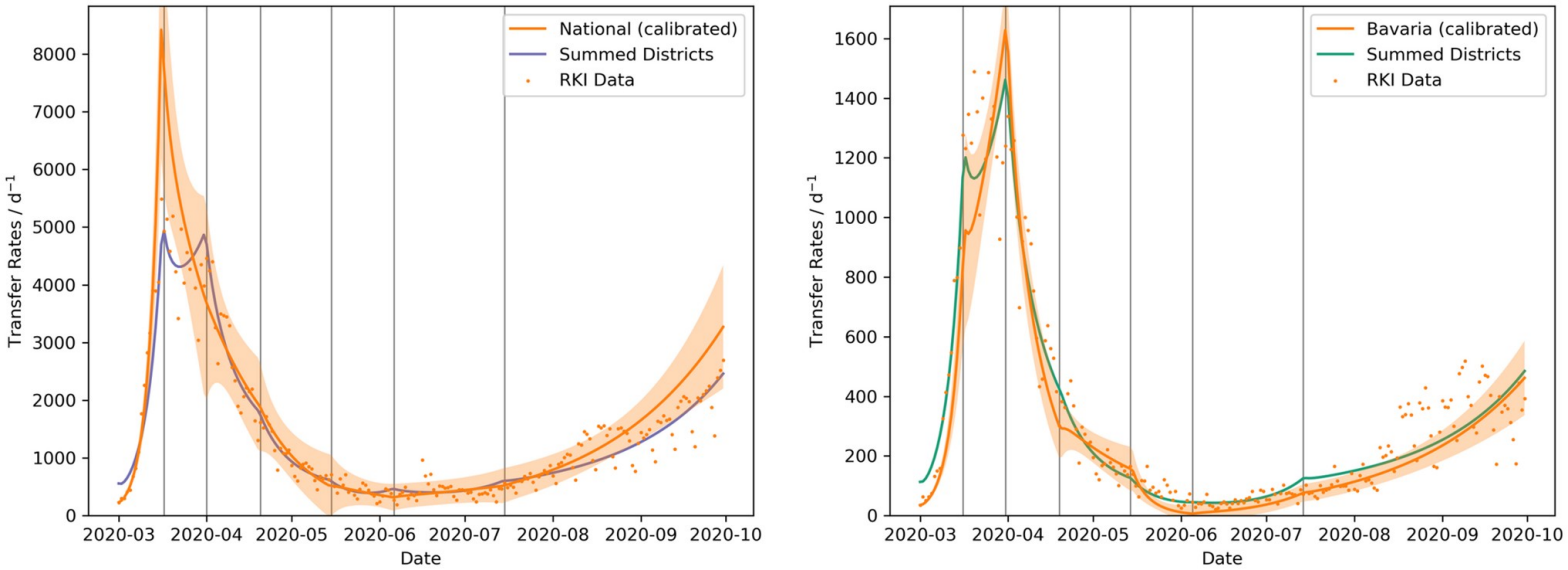
Comparison of fitted model results on different hierarchical levels. Comparisons of parametrized model runs on a higher hierarchical level with the aggregated case numbers from the fitted district level models. The fitted national model and the summed positive cases resulting from the 412 district level models are compared to the nationwide reported cases in the left panel and the fitted state model of Bavaria and the summed positive cases resulting from its 96 district level models are compared to the reported cases in Bavaria in the right panel. The shaded area indicates the 95% credible interval. The vertical grey lines indicate the dates of the NPIs.

The aggregated and nationally calibrated approaches are compared to the German-wide positively tested cases over time (Fig 3). First of all, it can be seen that the calibrated national SEIRD-model (1)–(5) with the variable contact rates can be used to reproduce the epidemic in Germany. Aggregating the simulation results from the fitted district models also reproduces the case numbers on a national level, but with some interesting deviations from the fitted national model. The very fast increase of reported cases until mid of March is matched well by both approaches. The subsequent peak is underestimated by the aggregated models. At the beginning of April, they show a second peak, which does not appear in the national model. For lower infection rates, the accumulated models perform well, although they tend to show minor peaks at the NPI change points. From the final NPI on, the spreading events become more scattered with a higher variance and the aggregated models underestimate the case numbers. There is a problem with the initial conditions, because at the early stage of the epidemic, many districts did not have any reported cases or had larger periods with zero infections. Therefore, the cases have to be integrated over several days for non-trivial initial conditions. This causes the aggregated cases to be larger at the start of the simulation.

Similarly and very easily within this modeling framework, the district level data can be aggregated to the next hierarchical level, namely the states. As an example, the state of Bavaria, which had the most cases of all German states during the first wave, is taken. The result is similar to the comparison of the national model. The aggregated reported cases show two peaks, whereas the state model only shows one late peak. The peaks at the dates of the NPIs are also present and the aggregated models underestimate the slow and scattered increase from August on.

**Fig 4:**
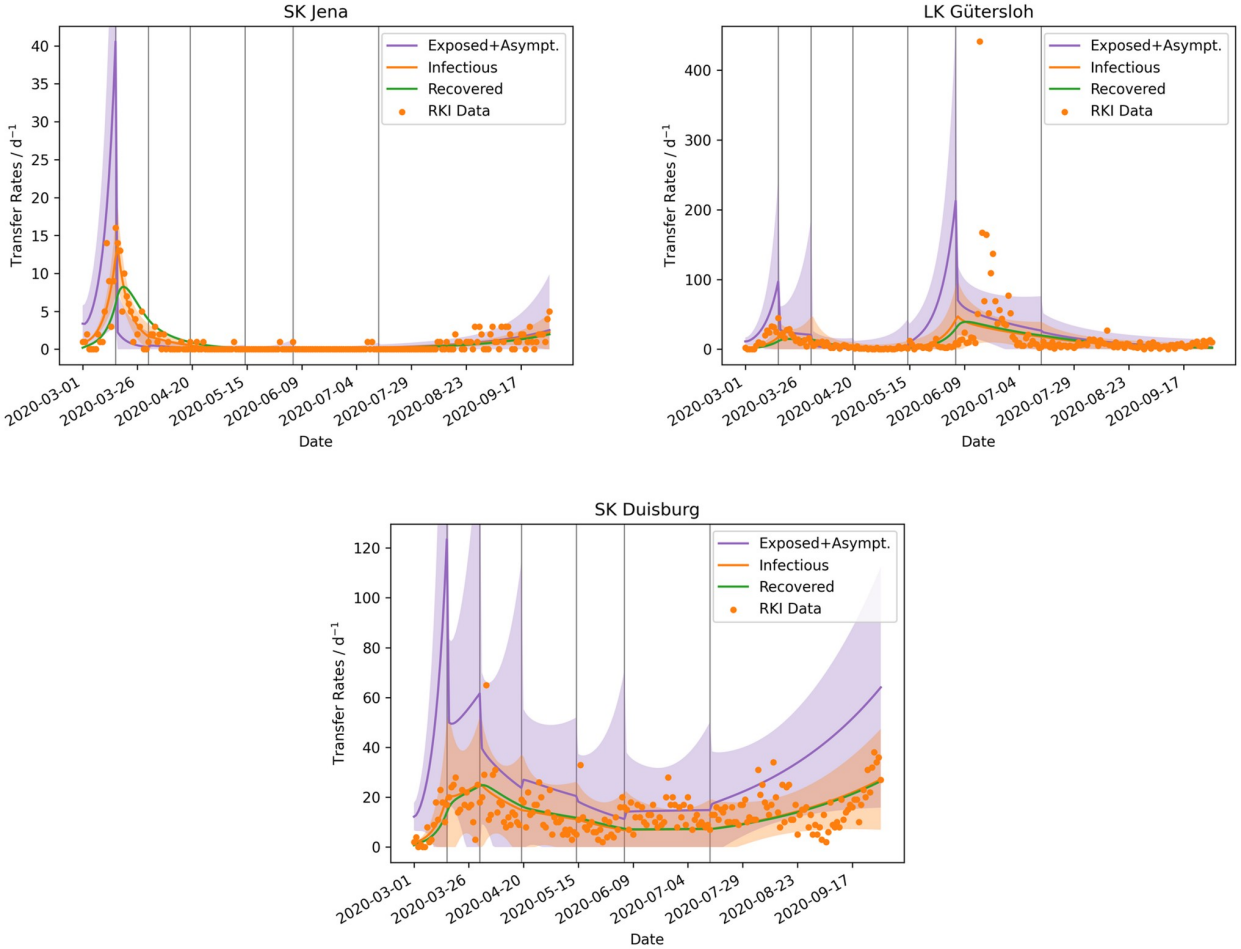
Time evolution of different transfer rates in three districts. The transfer rates into the compartment *Exposed* $\left(\frac{\beta_{j}}{N}IS\right)$ is shown in purple, into *Infectious* (*αE*) in orange, and into *Recovered* (*κE* + *γI*) in green. The shaded area shows the 95% credible interval of the rates. The reported positive cases are shown as a scatter plot in orange, corresponding to $I_{\text{obs}}\;\hat{=}\;\alpha E$. The vertical grey lines indicate the dates of the NPIs.

Now that we have seen that the aggregated fitted simulations can reproduce the reported case numbers on higher hierarchical levels, we can analyse individual districts and see what is being averaged out, when looking at the case numbers on a higher hierarchical level. At the same time, the capabilities and limits of the modified SEIRD model (1)–(5) applied to districts are shown. The results of the parametrized simulations for three districts with qualitatively different courses of the epidemic are discussed here. The results of the model runs fitted to the Stadtkreis (SK, urban district) Jena, Landkreis (LK, rural district) Gütersloh, and SK Duisburg, respectively (Fig 4) are presented now.

**Fig 5:**
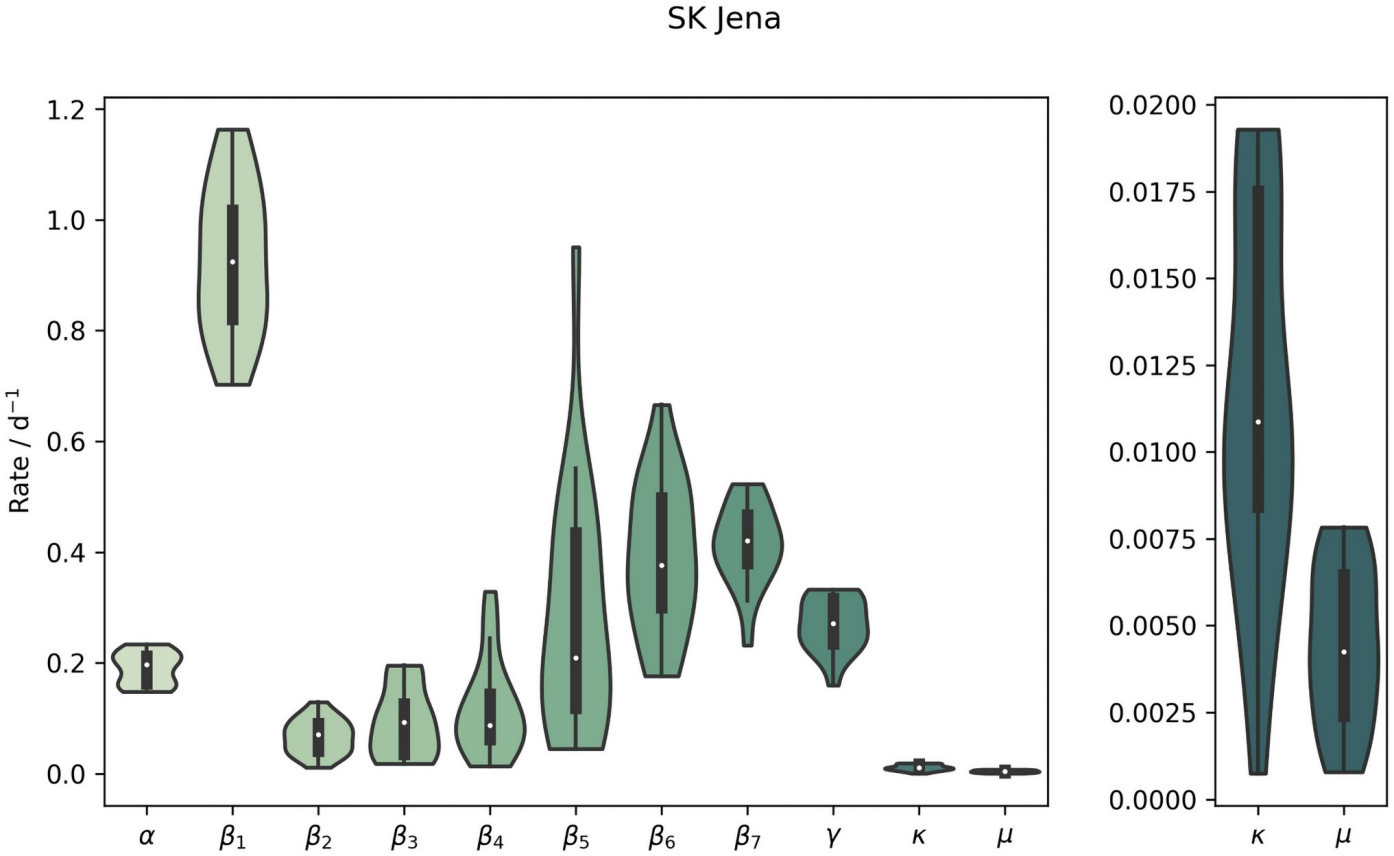
Posterior parameter distributions for SK Jena. The posterior distributions of the parameters for SK Jena. For better visualization, the parameters *κ* and *μ* are shown again on a separate y-scale. A classical box plot is show inside the violins, with the white dot indicating the optimal parameter.

SK Jena was the first district to introduce mandatory mask-wearing and at the same time, this district was very successful in quickly reducing the confirmed cases to almost zero, with only a few days over several month when single new cases were confirmed. This reduction might be a direct consequence of the mandatory face masks [7]. The drop in cases can also be seen from the fitted model results, where the peak of the newly reported cases was around the time the first NPI was implemented. After this peak, the rate quickly decreased to around zero per day at the time of the third NPI. The gradual increase of uncertainty in the contact rates from *β*_2_ to *β*_6_ is a result of the very low case numbers (Fig 5).

Compared to Jena, LK Gütersloh had a broader peak of infections at the beginning of the epidemic, but at the time of the third NPI, the rate became very low here too. This changed in mid June when a major outbreak occurred at a meat processing plant, with over 1000 infected employees [8, 9]. This outbreak was spread out over LK Gütersloh and LK Warendorf, where many of the employees lived. This outbreak lasted about two weeks, but the model spreads and broadens the peak between the NPI change points before and after the event. This is an issue of the insufficient temporal resolution of the contact rates *β*_*j*_. A drawback of the current parameter estimation is revealed by the model results for Gütersloh. The estimation of all contact rates *β*_*j*_ is done simultaneously and not for each NPI period successively. This problem arises before the fifth NPI, where the number of exposed and infectious individuals increases only to decrease after the NPI in order to match the data better.

SK Duisburg has had a mean infection rate of ${\bar{I}}_{\text{obs}}=14\;{\text{d}}^{-1}\pm 8\;{\text{d}}^{-1}$ with a standard deviation of 58% without a significant trend. Linearly fitting the data results in a slope of only $\frac{d{\bar{I}}_{\text{obs}}}{dt}=0.016\;{\text{d}}^{-2}$. Although SIR-type models tend towards either an exponential increase or decrease of the rates, the modified SEIRD model (1)–(5) reproduces the linear trend in Duisburg satisfactorily. The high variance of the reported cases affects the 95% credible interval, where the spread is much higher relatively to the two other analysed districts.

We have performed G-tests [26] to assess the goodness of fit for all models. Except for districts where only single-digit infection rates occur in intervals of up to several weeks, like LK Altmarkkreis Salzwedel, the models all reproduce the observed infection and death rates with a high probability (*p* < 0.05).

**Fig 6:**
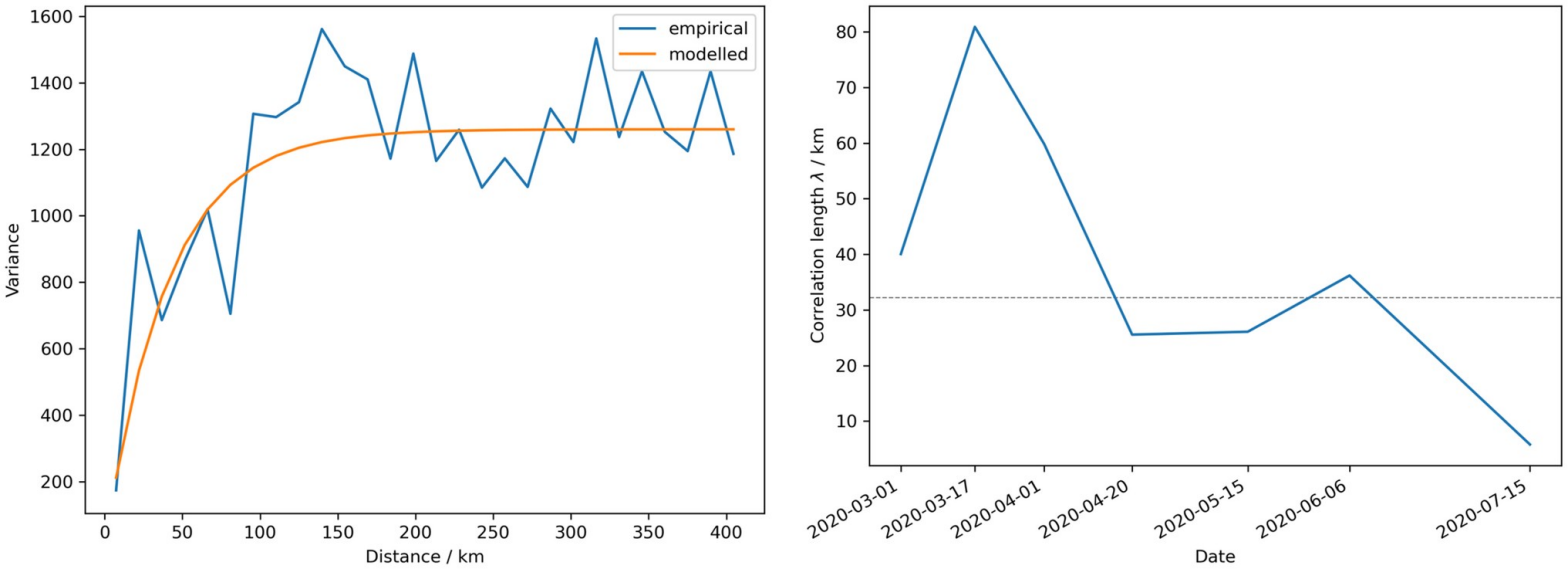
Results from the variogram analyses. The empirical and the exponential variograms Eq (9) of the cumulative rates of the reported cases for the time period before the first NPI are shown in the left panel. The time evolution of the correlation lengths λ_*i*_ of the covariance models for the cumulative cases is shown in the right panel. The mean distance of the neighboring district centroids is indicated by the dashed grey line.

A different view of the course of the epidemic can be gained by looking at the variograms of the infection rates. The variogram and its fit for a single NPI period from 2020–03-17 until 2020–03-23 of the cumulative case rates are shown in Fig 6. The variograms for all periods can be found in the appendix (S2.3 Fig in S1 File). The correlation lengths, derived from the variograms, increase from about λ_1_ = 40 km and peak at the crest of the first wave at twice the length λ_2_ = 81 km, when the first NPIs where implemented (Fig 6). From then on, the correlation lengths drop until the first NPIs are relaxed on 2020–04-20 with λ_4_ = 26 km, where the correlation lengths stay nearly constant until a minor peak at λ_6_ = 36 km is reached. Finally, a global minimum of λ_7_ = 5.8 km is reached with the last relaxation of the NPIs. For comparison, the district centroids have a mean distance to their neighboring district centroids of about 32 km.

## Discussion

In this work, we present a modified SEIRD-type epidemiological model with variable contact rates tailored to the COVID-19 pandemic. This model is fit to the data from each of the 412 German districts, all 16 states, and the nation. The parametrization is done using RKI data of the daily positively tested cases and the COVID-19 related deaths. The most important tool to modulate the epidemic to date, the non-pharmaceutical interventions, are implemented using piecewise constant contact rates which only change at the dates of NPI implementations. This model is flexible enough to satisfactorily reproduce the time evolution of the epidemic on a district level over many months, although the development of the epidemic is qualitatively very different across the different districts. Some districts had a very pronounced first peak followed by a long period in which the disease was practically eradicated. Others had a more or less constant rate of positively tested cases over several months. Furthermore, the same model can reproduce the epidemic on a state and on a national level. However, only on the district level is the spatial resolution high enough to analyse spatial patterns, for which we use the geostatistical method of variogram estimation. This method does not require any additional data, which makes variogram analysis an ideal tool during the onset of new epidemics, when only limited data are available.

Monitoring and modeling the infections on this small scale level is a first step towards local, precise, and target-oriented NPIs. Doing so could increase the cost/benefit ratio and also the acceptance of NPIs. The correlation lengths of the estimated variograms might help in evaluating if local NPIs are sufficient or if state or even nationwide measurements should be taken. An example scenario where the case numbers or weekly incidence rates alone are not enough to judge the effectiveness of local-scale NPIs is the following. If the quarantining in the aftermath of a superspreader event is applied too late or not rigorously enough, it could reduce the total amount of newly reported cases, but commuters might have already spread the disease to neighboring districts. In these surrounding districts, the case numbers would only slowly increase. Thus, by only taking the total case numbers into account, one might come to the conclusion, that the superspreader event was successfully quarantined. Whereas the correlation length would increase early with the slow spreading to the neighboring districts, even though the total amount of reported cases drops after the initial quarantining. This information can also be extracted from maps, but they contain the information in complex ways and it is always easier to communicate information in single numbers (e.g. weekly incidence rates, instead of the time evolution of the reported cases, the mean instead of the complete distribution, the *h*-index instead of the quality and topics of a researcher).

The high spatial resolution of the district level opens up the possibility to aggregate the results to a specific level, e.g. to the states or to the national level, which can also yield unique insights into the epidemic. The aggregated district models show a second peak during the first wave on 2020–04-01 (Fig 3). This might actually hint at the large number of districts, where the peak infection was reached with a delay of about two weeks compared to the districts, in which the epidemic started earlier. On a national level this delay is completely averaged out and it cannot be seen in the data on a German-wide level. Later on, the aggregated district models tend to underestimate the national-level case numbers. A reason for this could be that the dynamics of the epidemic are often driven by local superspreader events, which could be isolated and quarantined effectively. These events look like outliers on a district level, but increase the averaged cases on a national level, making them easier to match on the higher level. From August on, the infections seem to become more scattered with a much higher variability than before. This is also roughly the time, when more local NPIs were implemented and a central modeling approach with fixed NPIs for all districts might become too rigid for this kind of scenario.

The correlation lengths λ_*i*_ obtained from the variogram estimation support the idea that districts are the appropriate level of granularity for monitoring and modeling the epidemic. The fact that exponential variograms fit the data best further supports this, as it is a relatively rough correlation type, compared to e.g. Gaussian variograms, indicating that although pronounced spatial correlations exist, immediately neighboring districts can still have very different case numbers. If λ_*i*_ is less than the average neighboring district distance, it indicates that NPIs should only be implemented on a local district level, according to e.g. the weekly incidence rates of the district, published by the RKI. However, λ_*i*_ greater than the inter-district distance and less than the average distance between neighboring states suggests that NPIs should be applied on a state level or on an intermediate level, e.g. in Regierungsbezirken (provinces) in Germany. If the clusters grow beyond state size, nationwide NPIs are likely to be appropriate again. This hierarchical control approach works in both directions, not only for applying new NPIs at targeted spatial extents, but also for lifting existing ones over different regions, as the epidemic subsides. This modeling framework also makes it very easy to make projections on different hierarchical levels, e.g. what effect would NPIs have on the weekly incidence rates, if they are applied locally at a district level or if they are applied on a state level. Combining this with an economic model could help finding a balance between the effectiveness and costs of NPIs. It should be kept in mind, that the case numbers we calibrate the model against are likely to be underreported. A seroepidemiological study in four especially affected areas in Germany found between 1.6 and 6 times more infections than officially reported [27]. As a consequence, the reports are a lower boundary of the actual number of newly infected individuals. However, this lower boundary can still be used as a proxy for future intensive care unit demands [6]. However, if the testing strategies do not change considerably, there is no reason to believe that the relative changes in the actual occurrences of infections do not strongly correlate with the reported ones. Consequently, the effects of NPIs can be estimated, as they influence the time derivative of the reported cases and thus the relative change. The death rates seem to be more reliable and it was even suggested that they are a better metric for comparing the pandemic across different countries than infection rates [28].

The model results will likely improve, if the NPI periods are parametrized individually and successively. This would prevent the model from increasing the number of cases prior to an NPI and the actual increase, as can be seen in the results for LK Gütersloh at 2020–06-09 (Fig 4) or in the peaks at the NPI dates in the aggregated models (Fig 3). However, a multitude of approaches for such a successive parametrization exist. The approach presented in this study could be a precursor from which all constant parameters (*α*, *γ*, *κ*, *μ*) are identified. Subsequently, the contact rates *β*_*j*_ could be parametrized successively by regarding one NPI period at a time and with priors for *β*_*j*_ taken from the precursor run. Alternatively, the constant parameters could also be estimated for each NPI period separately. The differences in these supposedly constant parameters could be used as an indicator, to see if the compartments should be further divided into different age groups, as these parameters do vary between different age groups. But exploring these possibilities is beyond the scope of this work.

A further and likely more important improvement might be to choose an appropriate algorithm out of the wealth of published outlier detection algorithms [29] and to apply it to the RKI time series to automatically identify superspreader events. Such an event could then be implemented into the existing modeling framework by means of an additional transfer term, which acts like a Dirac pulse type source term for the *Infectious* compartment, but at the same times obeys the conservation laws. This way, local NPIs can be detected automatically and applied without having to prescribe NPIs manually to all districts individually.

An alternative approach could be to derive information about superspreader events from identifying change points in the contact rates as done by Dehning et al. [30].

SEIRD-type models are used for predictions or scenario modeling [6, 31]. Evaluating the predictive capabilities of the modified SEIRD model (1)–(5) is thus a future direction of our work. This could be combined with the outlier detection in order to automatically raise a flag in case of a sudden increase in newly reported cases over the past few days.

## Supporting information

S1 Fig Two examples of spatial random fields and their variograms. Borders republished from [32] under a CC BY licence, with permission from GeoBasis-DE / BKG, original copyright 2019. © GeoBasis-DE / BKG (2021).
(PNG)

S1 File Model assessment. (PDF)

## References

[1] Cucinotta D, Vanelli M. WHO Declares COVID-19 a Pandemic;91(1):157–160. doi: 10.23750/abm.v91i1.9397 PMC7569573 32191675

[2] Ferguson N, Laydon D, Nedjati Gilani G, Imai N, Ainslie K, Baguelin M, et al. Report 9: Impact of non-pharmaceutical interventions (NPIs) to reduce COVID19 mortality and healthcare demand;. Available from: http://spiral.imperial.ac.uk/handle/10044/1/77482.

[3] Khailaie S, Mitra T, Bandyopadhyay A, Schips M, Mascheroni P, Vanella P, et al. type [;]Available from: http://medrxiv.org/lookup/doi/10.1101/2020.04.04.20053637. 10.1186/s12916-020-01884-4 PMC7840427 33504336

[4] Nicola M, Alsafi Z, Sohrabi C, Kerwan A, Al-Jabir A, Iosifidis C, et al. The socio-economic implications of the coronavirus pandemic (COVID-19): A review;78:185–193. doi: 10.1016/j.ijsu.2020.04.018 PMC7162753 32305533

[5] Statistisches Bundesamt [Destatis];. Available from: https://www.destatis.de/EN/Themes/Economy/National-Accounts-Domestic-Product/Tables/gdp-bubbles.html.

[6] Barbarossa MV, Fuhrmann J, Meinke JH, Krieg S, Varma HV, Castelletti N, et al. Modeling the spread of COVID-19 in Germany: Early assessment and possible scenarios;15(9):e0238559. doi: 10.1371/journal.pone.0238559 PMC7473552 32886696

[7] Mitze T, Kosfeld R, Rode J, Wälde K. Face Masks Considerably Reduce COVID-19 Cases in Germany: A Synthetic Control Method Approach; p. 31. 10.1073/pnas.2015954117 PMC7768737 33273115

[8] Guenther T, Czech-Sioli M, Indenbirken D, Robitailles A, Tenhaken P, Exner M, et al. Investigation of a superspreading event preceding the largest meat processing plant-related SARS-Coronavirus 2 outbreak in Germany; doi: 10.2139/ssrn.3654517

[9] RKI—Homepage;. Available from: https://www.rki.de/EN/Home/homepage_node.html.

[10] Kergaßner A, Burkhardt C, Lippold D, Kergaßner M, Pflug L, Budday D, et al. Memory-based meso-scale modeling of Covid-19: County-resolved timelines in Germany;66(5):1069–1079. doi: 10.1007/s00466-020-01883-5 32836600 PMC7398641

[11] Kapoor A, Ben X, Liu L, Perozzi B, Barnes M, Blais M, et al. Examining COVID-19 Forecasting using Spatio-Temporal Graph Neural Networks;.

[12] Wang L, Xu T, Stoecker TH, Stoecker H, Jiang Y, Zhou K. Machine learning spatio-temporal epidemiological model to evaluate Germany-county-level COVID-19 risk;.

[13] Statistisches Bundesamt [Destatis];. Available from: https://www.destatis.de/DE/Themen/Laender-Regionen/Regionales/Gemeindeverzeichnis/Administrativ/04-kreise.html.

[14] Kermack WO, McKendrick AG. A Contribution to the Mathematical Theory of Epidemics;115(772):700–721.

[15] Diekmann O, Heesterbeek JAP, Roberts MG. The construction of next-generation matrices for compartmental epidemic models;7(47):873–885. doi: 10.1098/rsif.2009.0386 PMC2871801 19892718

[16] Dormand JR, Prince PJ. A family of embedded Runge-Kutta formulae;6(1):19–26. doi: 10.1016/0771-050X(80)90013-3

[17] SciPy 1 0 Contributors, Virtanen P, Gommers R, Oliphant TE, Haberland M, Reddy T, et al. SciPy 1.0: fundamental algorithms for scientific computing in Python;17(3):261–272. doi: 10.1038/s41592-019-0686-2 PMC7056644 32015543

[18] Metropolis N, Rosenbluth AW, Rosenbluth MN, Teller AH, Teller E. Equation of State Calculations by Fast Computing Machines;21(6):1087–1092. doi: 10.1063/1.1699114

[19] Hastings WK. Monte Carlo sampling methods using Markov chains and their applications;57(1):97–109.

[20] Houska T, Kraft P, Chamorro-Chavez A, Breuer L. SPOTting Model Parameters Using a Ready-Made Python Package;10(12):e0145180. doi: 10.1371/journal.pone.0145180 PMC4682995 26680783

[21] Schnicke T, Langenberg B, Schramm G, Krause C, Strempel T. EVE - High-Performance Computing Cluster;.

[22] Saltelli A, Tarantola S, Chan KPS. A Quantitative Model-Independent Method for Global Sensitivity Analysis of Model Output;41(1):39–56. doi: 10.1080/00401706.1999.10485594

[23] Matheron G. Principles of geostatistics;58(8):1246–1266. doi: 10.2113/gsecongeo.58.8.1246

[24] Rubin Y. Applied Stochastic Hydrogeology. Oxford University Press;.

[25] Schüler L, Müller S. GeoStat-Framework / GSTools: Volatile Violet v1.2.1;. Available from: https://zenodo.org/record/3751743.

[26] Sokal RR, Rohlf FJ. Biometry: the principles and practice of statistics in biological research. 3rd ed. W.H. Freeman;.

[27] Santos-Hövener C, Busch MA, Koschollek C, Schlaud M, Hoebel J, Hoffmann R, et al. Seroepidemiological study on the spread of SARS-CoV-2 in populations in especially affected areas in Germany—Study protocol of the CORONA-MONITORING lokal study;. 10.25646/7053 PMC8734078 35146295

[28] Lau H, Khosrawipour T, Kocbach P, Ichii H, Bania J, Khosrawipour V. Evaluating the massive underreporting and undertesting of COVID-19 cases in multiple global epicenters;27(2):110–115. doi: 10.1016/j.pulmoe.2020.05.015 PMC7275155 32540223

[29] Hodge VJ, Austin J. A Survey of Outlier Detection Methodologies;22:85–126. doi: 10.1023/B:AIRE.0000045502.10941.a9

[30] Dehning J, Zierenberg J, Spitzner FP, Wibral M, Neto JP, Wilczek M, et al. type [;]Available from: http://medrxiv.org/lookup/doi/10.1101/2020.04.02.20050922. 10.1126/science.abb9789 PMC7239331 32414780

[31] Maier BF, Brockmann D. Effective containment explains subexponential growth in recent confirmed COVID-19 cases in China; p. eabb4557. doi: 10.1126/science.abb4557 PMC7164388 32269067

[32] GeoBasis-DE / BKG 2019;. Available from: https://gdz.bkg.bund.de/index.php/default/open-data/verwaltungsgebiete-1-250-000-ebenen-stand-31-12-vg250-ebenen-31-12.html.

