# Supplementary figures and images for: Data driven high resolution modeling and spatial analyses of the COVID-19 pandemic in Germany
Source: PLoS One. 2021 Aug 18;16(8):e0254660. doi: 10.1371/journal.pone.0254660 (PMC8372931; doi:10.1371/journal.pone.0254660)

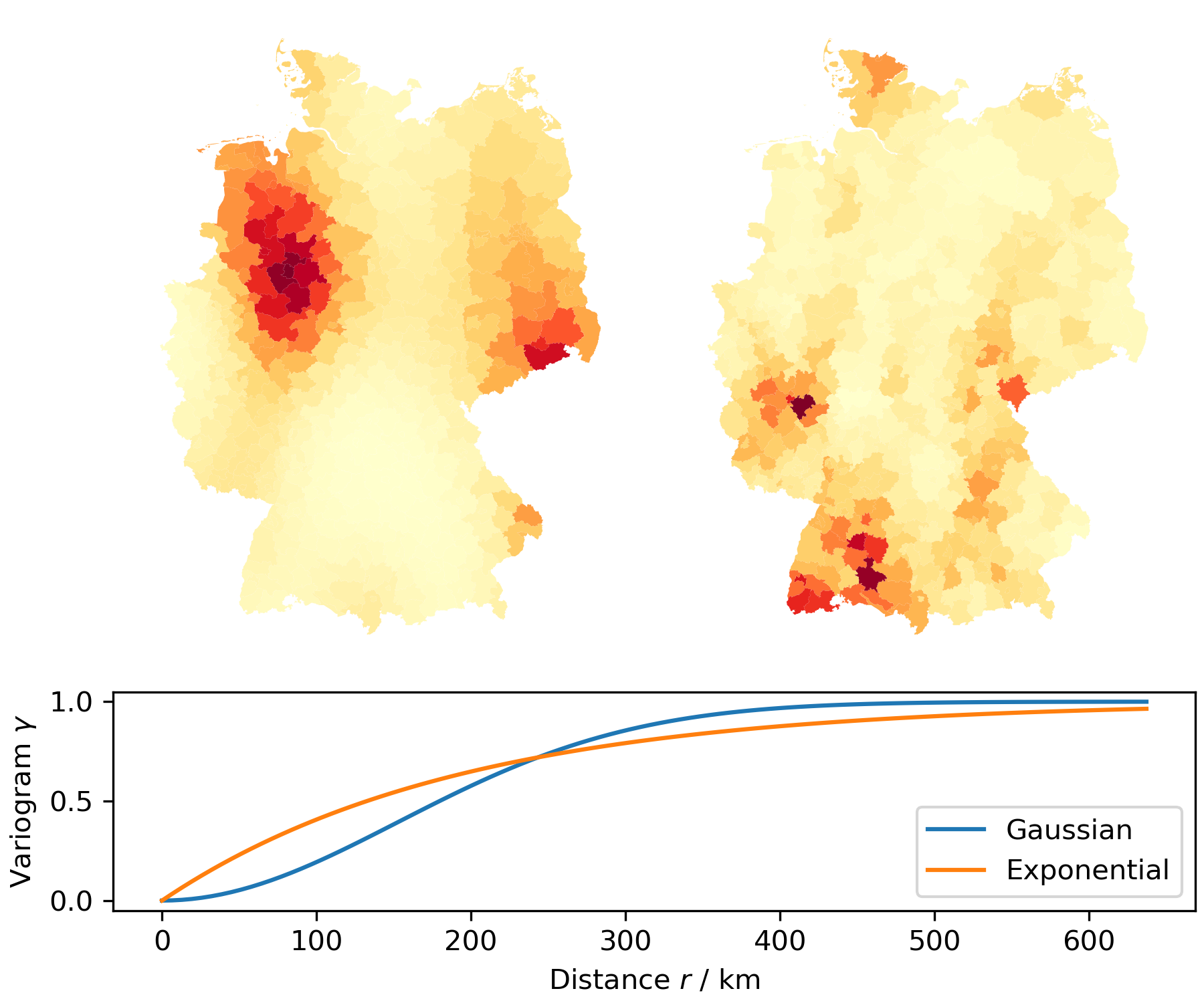

Supplement: S1 Fig — Borders republished from [32] under a CC BY licence, with permission from GeoBasis-DE / BKG, original copyright 2019. © GeoBasis-DE / BKG (2021). (PNG) [file pone.0254660.s001.png]
