# Supplementary material for: Data driven high resolution modeling and spatial analyses of the COVID-19 pandemic in Germany
Source: PLoS One. 2021 Aug 18;16(8):e0254660. doi: 10.1371/journal.pone.0254660 (PMC8372931; doi:10.1371/journal.pone.0254660)
Supplement: S1 File — (PDF) [file pone.0254660.s002.pdf]

S2 Appendix. Data driven high resolution modeling and spatial analyses of the COVID-19 pandemic in Germany - Model assessment

Authors: Lennart Schüler, Justin M. Calabrese, Sabine Attinger

With the 412 districts simulated with fitted models, we can create histograms of the model parameters. Looking at the distributions of the model parameters across the districts, it is to be expected that mostly the contact rates β_j should vary across districts (Fig S2.1). Except for some variations in the age structures of the populations, the other model parameters should not vary strongly. But this is only the case for the recovery rate γ, which has a pronounced peak at about $\gamma \approx 3.2\,\mathrm{d}^{-1}$. The other three parameters are more or less uniformly distributed, but with a negative trend for α. The extended FAST sensitivity analysis (Fig S2.2) reveals that the three parameters α, κ, and μ are the least sensitive ones towards the calibrated data. Looking at the uniform distributions of κ and μ across the districts, they seem to be so insensitive that they cannot be uniquely identified, as the parameter calibration has no way of pinpointing them. From the low sensitivity one cannot deduce that the parameters are not important for the model, as the sensitivity analysis only tests the relative influence towards minimizing the objective function.

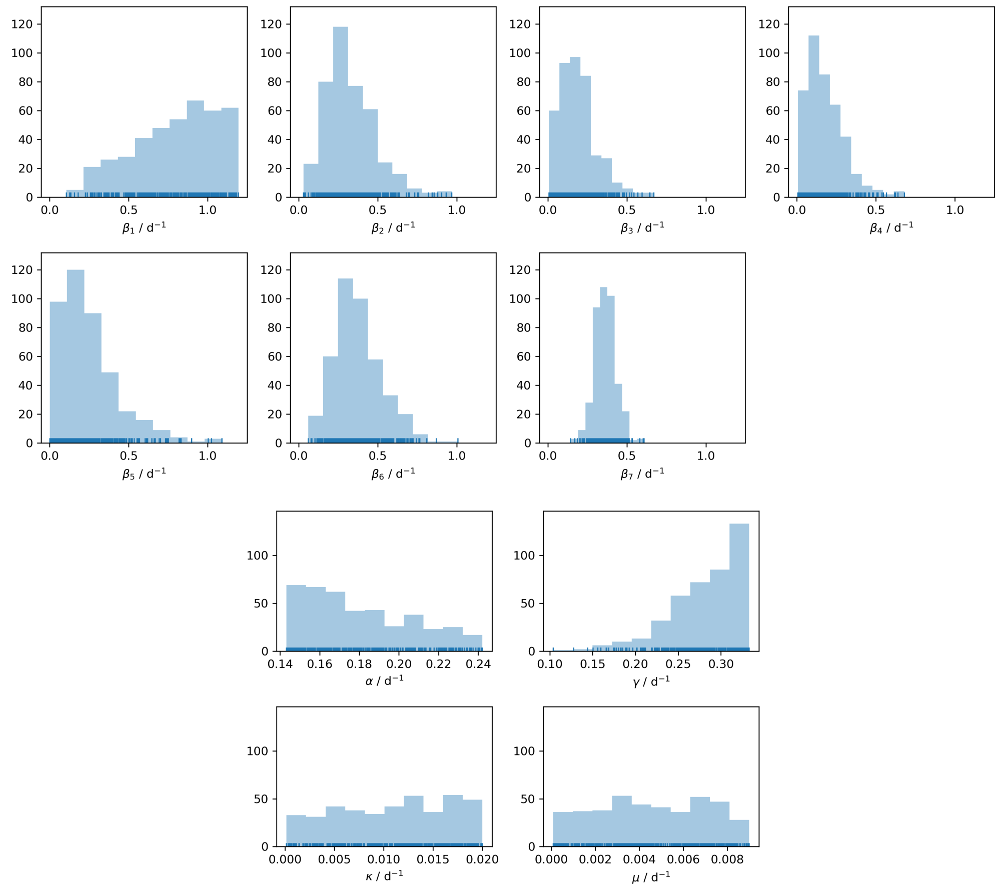

Fig S2.1. Histograms of the parameters from all 412 districts. The rug plot indicates each single parameter value with a small vertical tick.

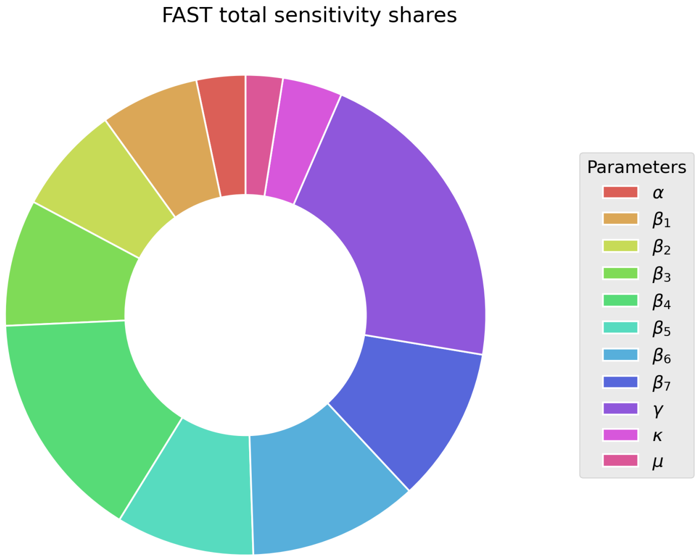

Fig S2.2. Parameter sensitivity for SK Duisburg. The relative sensitivities of each parameter exemplarily for SK Duisburg. The larger the slice of a parameter, the more it influences the simulation results in regard to the observations, which are the positively tested case rate and the COVID-19 related death rate.

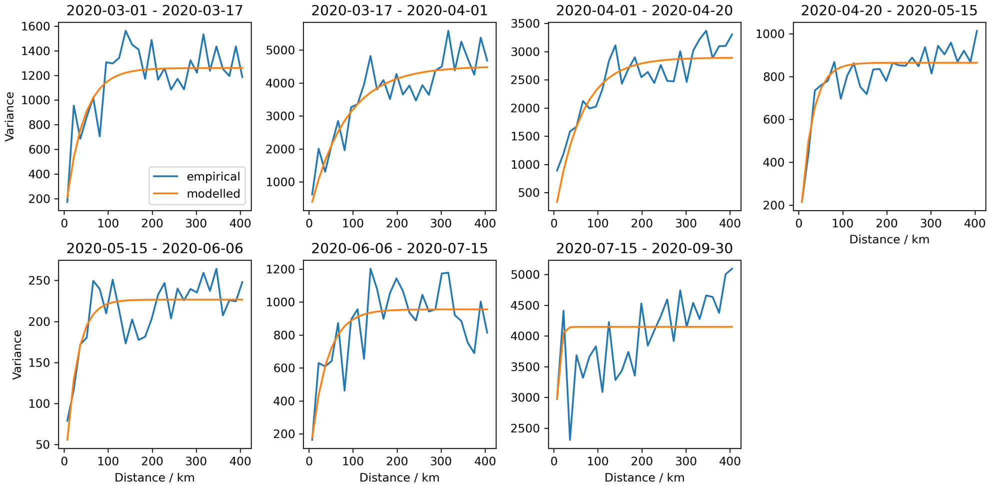

Fig S2.3. Variograms of the reported cases over different time periods. The empirical and the modelled exponential variograms of the cumulative rates of reported cases for every NPI period. The variance is proportional to the cases and the flattening of the exponential variograms indicates the correlation length.
